# Percutaneous Nephrolithotomy in Children

**DOI:** 10.1155/2011/123606

**Published:** 2011-10-13

**Authors:** Romano T. DeMarco

**Affiliations:** Departments of Surgery and Pediatrics, Division of Pediatric Urology, Sanford Children's Hospital, 1600 W. 22nd Street, Sioux Falls, SD 57104, USA

## Abstract

The surgical management of pediatric stone disease has evolved significantly over the last three decades. Prior to the introduction of shockwave lithotripsy (SWL) in the 1980s, open lithotomy was the lone therapy for children with upper tract calculi. Since then, SWL has been the procedure of choice in most pediatric centers for children with large renal calculi. While other therapies such as percutaneous nephrolithotomy (PNL) were also being advanced around the same time, PNL was generally seen as a suitable therapy in adults because of the concerns for damage in the developing kidney. However, recent advances in endoscopic instrumentation and renal access techniques have led to an increase in its use in the pediatric population, particularly in those children with large upper tract stones. This paper is a review of the literature focusing on the indications, techniques, results, and complications of PNL in children with renal calculi.

## 1. Introduction

Following the first report of percutaneous stone surgery for upper tract stones in 1976 [1], the use and application of PNL has increased dramatically. While randomized studies comparing the efficacy of PNL to other forms of therapy are lacking, it is commonly employed in adults with large renal stones and is the recommended treatment for adults with staghorn calculi [2]. 

The first pediatric series evaluating the use of PNL in children was reported by Woodside and associates in 1985 [3]. Following this report, the acceptance of PNL as being a safe and effective therapeutic option in children was slow. However, over the last decade with advances in access techniques and instrumentation, PNL has replaced open surgery and is an alternative to SWL and ureteroscopy (URS) in those pediatric patients with large upper tract calculi. In certain patients, PNL appears to be a better option than either SWL or URS. This paper provides a review of the literature, focusing on recent advances in the use of PNL for treating large upper tract stones in children. 

## 2. Discussion

### 2.1. Indications

After its acceptance as a safe and effective therapy in children, PNL was initially reserved as a secondary procedure in those children who had failed SWL or was used as part of sandwich therapy with SWL. More recently, it has been used as monotherapy. Indications for PNL in children are similar to those in adults. These general indications include stone burden greater than 1 cm, complete or partial staghorn calculi, lower pole stone, anatomic abnormalities which may impede urinary drainage and clearance, and known or suspected struvite or cysteine stones [4]. Erdenetsesteg et al. reported their success with various stone treatments and reported a stone-free rate of 96% in those children who underwent PNL for the following: staghorn calculus, stone >2 cm, gross hydronephrosis, poorly functioning kidney, infected hydronephrosis or pyonephrosis, or upper ureteral stone >1 cm [5].

### 2.2. Instrumentation

Following the advent of PNL, its use in children was limited primarily because of the concerns of renal damage due to the size of the renal access and/or the adult-sized instruments [6]. Recent advances in renal access techniques and miniaturization of endoscopic instruments have allowed for limited tract dilation. Standard rigid nephroscopes range in size from 19.5 to 27 Fr and were employed routinely until the introduction of a 17 Fr pediatric nephroscope in 1989 [7]. Further refinements in technology have led to the development of 15/18 Fr rigid nephroscope. If needed, additional miniaturization can be obtained by downsizing to a rigid cystoscope (7 F).

Flexible endoscopy can be performed with a standard 15 Fr nephroscope or smaller, shorter 11 Fr pediatric cystoscopes, or 7 to 9 Fr ureteroscopes. Restrictions are encountered with smaller endoscopes, as they limit visualization because of the small working and irrigating channels. Flexible ureteroscopes are long and can be cumbersome when working in the renal pelvis or calices. 

The choice of lithotriptor is generally left up to the individual surgeon. Ultrasonic or pneumatic lithotriptors have been primarily used. These machines are very efficient for stone removal, as they can provide both lithotripsy and evacuation of stone fragments via suction. The holmium:YAG laser is an alternative energy source and is particularly useful when a small access sheath is required [4].

### 2.3. Renal Access

#### 2.3.1. General Concepts

The placement of percutaneous access into the collecting system is arguably the most critical aspect of a successful PNL [8]. Correct PNL access increases visualization and accessibility to the stone and decreases operative time. 

Understanding of basic renal anatomy is essential for gaining safe access. The avascular region between the anterior and posterior vessels of the kidney is known as Brodel's bloodless line and is the ideal area to enter the renal parenchyma. A posterior calix is the preferred site of entry, as it makes passage of wires past the UPJ easier and disrupts less renal parenchyma on entrance than an anterior calix. Puncture beyond the anterior aspect of the collecting system risks injury to the large anterior vessels which cannot be easily tamponaded with a balloon catheter. 

Sampaio et al. performed a 3-dimensional study of intrarenal anatomy using endocasts and determined the safest area to enter the kidney was the fornix [9]. In their study, entering directly into the fornix did not lead to arterial injury and had a low incidence of venous injury (<8%). However punctures through the infundibuli of the lower, middle, and upper poles were associated with vascular injury 68%, 38%, and 67% of the time, respectively. A direct forniceal strike also allows for smoother advancement of the access sheath into the calix and improves passage of instruments. Direct puncture of the renal pelvis should be avoided at all times, as it increases both the risk of large vascular injury and urine leakage. Renal access should be no further lateral than the medial border of the posterior axillary line to avoid colonic injury. Additionally, it is recommended that space be left between the access sheath and the overlying rib to avoid injury to the intercostal neurovascular bundle and limit postoperative nephrostomy tube discomfort.

In general, renal access for PNL is performed by interventional radiologists, with only 11% of urologists obtaining their own access [10]. Watterson et al. performed a retrospective review of patients at their institution who had access obtained by a urologist and compared these results to a group of patients who had access obtained by an interventional radiologist [11]. The group having a urologist gain access had a significantly improved stone-free rate and fewer access-related complications. Additional benefits may include a decrease in hospital stay and overall procedures, particularly in those institutions where access is done as a separate procedure in the interventional radiology suite. 

#### 2.3.2. Positioning

Following induction of anesthesia, the patient is placed in the dorsal lithotomy position for cystoscopy and placement of either an open-ended ureteral or balloon occlusion catheter for contrast opacification of the collecting system. A Foley catheter is placed, and the patient is then placed prone. A gentle roll is placed under the side with the stone to orient the posterior calices in a more direct parallel position for needle entry. A chest roll is placed to help with ventilation. The ipsilateral arm is placed in the swimmer's position with the opposite arm tucked at the side. Those patients with spina bifida and severe back curvature or chest wall deformities require unique consideration when positioning dependent on the individual's anatomy. Many will have Harrington rods or other orthopedic hardware which will interfere with fluoroscopy. It is best to position the child in such a way that the vertebral bodies closest to the desired access are aligned as close to normal as possible. This is best assessed with fluoroscopy, by having the transverse processes as close to a normal alignment during adjustments made with the C-arm in the parallel position.

#### 2.3.3. Access Techniques

**Figure 1:**
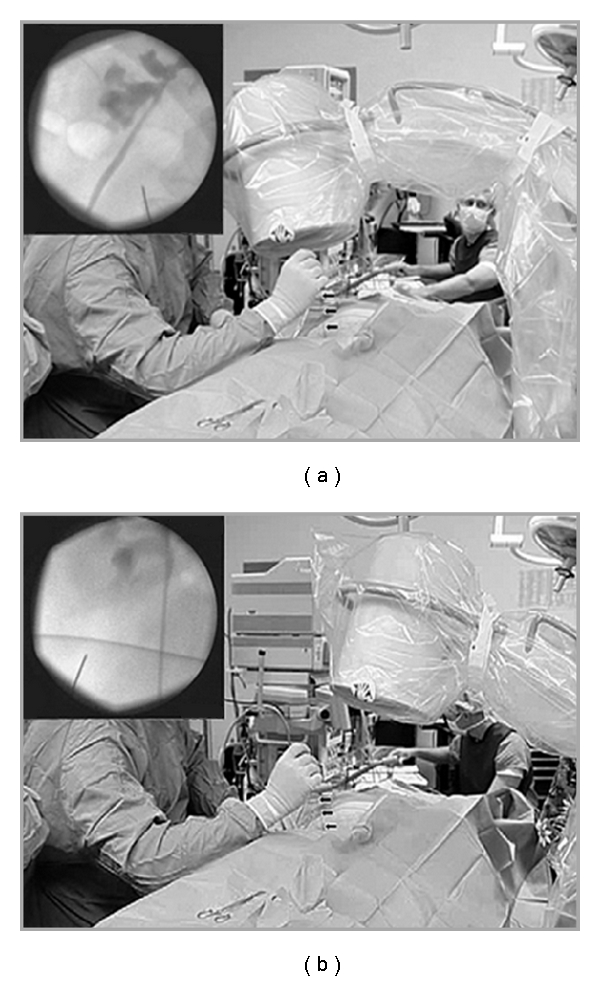
Triangulation technique for percutaneous access. Fluoroscopic C-arm is moved back and forth between 2 positions, including 1 parallel and 1 oblique to puncture line. A, with C-arm oriented parallel to puncture line, adjustments with access needle (arrows) are made in mediolateral (left/right) direction. Inset, corresponding fluoroscopic image. B, C-arm is rotated to oblique position, and adjustments with access needle are made in cephalad/caudal (up/down) orientation of puncture line. Inset, corresponding fluoroscopic image. (Reprinted with permission from The Journal of Urology, Vol. 178, Miller NL, Matlaga BR, and Lingeman JE. Techniques for Fluoroscopic Percutaneous Renal Access, pp 15–23, Copyright Elsevier 2007.)

While both ultrasound and CT guidance have been used for percutaneous renal access [12, 13], biplanar fluoroscopy is the most commonly used method. Two fluoroscopic techniques have been described. They include the “eye of the needle” and triangulation techniques (Figure 1). The triangulation procedure using biplanar fluoroscopy will be discussed. 

After the appropriate calix is chosen for entry, orientation of the line of puncture is performed by using the triangulation technique. Two positions, 1 parallel and 1 oblique to the line of puncture, are obtained with C-arm. With the C-arm parallel to the line of puncture, adjustments of the 18 gauge access needle are made in the left-right direction (mediolateral). Up-down (cephalad or caudal) movements are made when the C-arm is rotated to an oblique position. It is critical for the needle to be moved only in one plane when making adjustments to ensure proper orientation. This involves back and forth imaging in the two positions to make sure the needle stays on track. Full opacification of the collecting without extravasation is maintained via the retrograde catheter. After proper orientation of the line of puncture, ventilation is suspended in full expiration. Advancement of the needle is only performed with the C-arm in the oblique position, as this allows for gauging of needle penetration and depth. Prior to puncturing the renal capsule, further adjustments are made in both planes. Needle movement once the renal parenchyma is penetrated should only be done with the C-arm in the oblique position as transverse movement of the needle may move the kidney and alter the position of the calix. 

#### 2.3.4. Tract Establishment

Entrance into the collecting system is confirmed by the aspiration of urine. A hydrophilic glidewire is then advanced into the collection system, and attempts are made to advance the wire down the ureter. If unsuccessful, the wire can be coiled into the renal pelvis. An 8 Fr fascial dilator is then passed into the calix followed by a torqueable catheter to help guide the wire down the ureter. A ureteral catheter is then advanced over the wire, and the glidewire is then removed and a stiffer wire such as an Amplatz superstiff is placed. Over this, an 8 to 10 Fr coaxial catheter is advanced down the ureter, and a second safety wire is advanced and coiled in the bladder.

Tract dilation is then performed with either sequential or balloon dilators. While balloon dilators may be less traumatic and cause less bleeding than sequential dilators [14], sequential dilating is typically performed in the pediatric patient, as it affords the flexibility of choosing the desired sized sheath. A “mini-perc” technique described by Jackman and colleagues eliminates sequential dilating with placement of an 11 Fr peel-away access sheath [15]. When larger renal access is required both adult-sized sheaths (24 Fr) and balloon dilatation [16, 17] have been reported to be in safe in small children. 

### 2.4. Renal Drainage

At the conclusion of the procedure, placement of a nephrostomy tube is the standard of care. The choice of tube is up to the individual surgeon. Ten or 12 Fr loop nephrostomy tubes are typically placed after a relatively bloodless PNL. In those situations where more bleeding is encountered, a larger 12 or 14 Fr balloon catheter may be used to tamponade the bleeding. In older children and those situations when a secondary PNL for significant residual calculi will be performed, a 24 Fr re-entry Malecot catheter can be used (Table 1). Small reports of tubeless PNL have shown to be a safe option in children [18, 19].

### 2.5. Postprocedure Imaging

With the recent emphasis on radiation safety (ALARA principle) the use and choice of imaging study post-PNL is not standardized. The determination of which study is best becomes a balancing act of limiting radiation exposure while not compromising procedural success. Thorough endoscopic evaluation with mapping of each calix with a flexible nephroscope and fluoroscopy is mandatory in all patients at the end of the primary PNL and is specific for stone clearance [20]. Further imaging following PNL should be individualized and determined by the preoperative size and location of stone, the manner of stone removal, and findings at the time of the PNL. 

### 2.6. PNL Outcomes

#### 2.6.1. Stone-Free Rates

Reported stone-free rates range widely from 58% to 99% when PNL is used as primary, monotherapy (Table 2). Farhat and Kropp report a 100% stone-free rate in 22 children who underwent PNL from 1999 to 2005 with a secondary PNL in 8 and a tertiary procedure in 1 [4]. Similar results were noted by Salah and colleagues who reported a success rate of 99% following PNL in 135 children [21]. 

Factors such as age or heavy stone burden have not been found to affect outcomes. Samad et al. reported a stone-free rate of 67% in children <5 years but found only 49% of patients >5 to 16 years to have a successful PNL [32]. Unsal et al. reported a stone-free rate of 94% in patients younger than 7 years of age [33]. This group also noted no difference in stone-free rates in children with larger stone burdens. Similarly, Aron and colleagues found PNL monotherapy to be highly effective with stone-free rates approaching 90% in children aged 20 months to 5 years with staghorn calculi [22]. 

Little retrospective and no prospective data exists comparing success rates of PNL to other therapies. Shokeir and colleagues compared the results of 166 children who were treated with either PNL or SWL for 1 to 2 cm renal stones [34]. A stone-free rate of 87% was found in the group of patients who had undergone 1 PNL treatment. In comparison only 45% were stone-free following 1 SWL session. Similar outcomes were noted from the group at Vanderbilt Children's Hospital. They retrospectively compared outcomes in children with >1 cm renal calculi and noted a stone-free rate of 29% with 1 SWL session as compared to 100% in those who had PNL performed [35].

### 2.7. Complications

The most common serious complication following PNL is bleeding requiring transfusion. Zeren and colleagues reported significant intraoperative hemorrhage requiring transfusion in 24% of their patients [31]. On review of their patients, they found an association of transfusion with operative time, stone burden, and sheath size. They also postulated that the use of rigid nephroscopes and overlevering on the kidney may have led to increased bleeding. More recent studies have demonstrated much lower rates of transfusion (<5%) with an association between both tract number and size and need for transfusion [16, 27].

Other commonly reported complications include transient fever and urine leaks which required either prolonged nephrostomy tube drainage or ureteral stent placement [27]. Concerns regarding injury to the kidney following pediatric PNL have not borne out. Work done by Dawaba et al. demonstrated that at long-term followup, PNL in the pediatric population improved overall renal function without causing renal scarring [24]. 

### 2.8. PNL in Setting of UPJ Obstruction

In rare situations, PNL is performed in a child with a renal stone in the setting of a UPJ (ureteropelvic junction) obstruction. In this situation, the typical purpose is to gain a better assessment of renal function in a poorly functioning kidney after stone clearance. A complicating factor in this scenario is the possibility that injected contrast through the ureteral catheter will not opacify the intrarenal anatomy. In this rare situation, renal access can be gained by directly puncturing into a stone bearing calix. Careful injection of contrast into the calix can opacify the collecting system for puncture if another or different access is needed. Overzealous injection can lead to significant extravasation and should be avoided. If hydronephrosis is present, ultrasound-guided access can be obtained with subsequent refinement of the location of the access through antegrade contrast injection and subsequent calyceal puncture. 

Another alteration in gaining access and establishing a tract in this clinical setting is the need to “park” the guide wires in the kidney, as they will not typically advance down the ureter. The guide wires have less purchase in the kidney as compared to having the wires down the ureter and into the bladder. Because of this, the wires more easily can be dislodged or come out of the collecting system. In this situation, removal core guide wires are helpful, as they can be coiled more extensively into the kidney to prevent guide wire displacement.

### 2.9. Laparoscopic Pyelolithotomy

While PNL is arguably considered the primary therapy for children with large renal stones, laparoscopic pyelolithotomy is emerging as an alternative procedure. Both transperitoneal and retroperitoneal approaches have been utilized with good success in the adult population. Less has been reported in the use of this procedure in children. Casale and associates reported a stone-free rate of 87.5% using a transperitoneal approach in 8 children and recommended this approach be considered when percutaneous access is not feasible [36]. This approach is also supported by recent work by Skolarikos and colleagues, who on reviewing the level of evidence mainly in the adult population recommended laparoscopic nephro- or pyelolithotomy only in the clinical setting of a stone in an anterior diverticulum or when PNL or flexible ureteroscopy had failed [37]. 

## 3. Conclusions

Over the last 2 decades, refinements in percutaneous access techniques, miniaturization of instruments, and technologic advances in energy sources for lithotripsy have led to improved outcomes and lower morbidity rates in children following PNL. These factors have led to an increase in its use and its acceptance as standard therapy for children with large renal calculi. 

## Figures and Tables

**Table 1:** Standard products for percutaneous nephrolithotomy.

Opacification of the collecting system
(1) Open ended ureteral catheter either 5 Fr **OR**
(2) Occlusion balloon catheter 5 Fr

Puncture of calyx
(1) 18 gauge needle with either 12 or 20 cm introducer

Guidewire placement
(1) 0.035′′ guidewire with straight tip (first wire)
(2) 0.035′′ sensor guidewire with straight tip (first wire exchanged for this wire)
(3) 8/10 dilator sheath set
(4) 0.035′′ superstiff guidewire with straight tip (placed as second wire through 8/10)
(5) Torqueable catheters and angled glidewires and guidewires for difficult anatomy

Renal tract dilation/establishment
(1) Sequential renal dilation to desired sheath (internal diameter 12–24 Fr) **OR**
(2) High-pressure balloon dilation with placement of sheath (24–30 Fr)

Renal drainage following procedure
(1) Locking loop nephrostomy tubes 10–12 Fr **OR**
(2) Malecot re-entry catheters 16–24 Fr

**Table 2:** Comparison of pediatric percutaneous nephrolithotomy series DNS: did not specify; ^a^includes SWL therapy.

Study	No. children/renal units	Mean age (Yrs)	Mean stone size	Maximum sheath (Fr)	Multiple tracts %	Second look %	Stone-free initial/final %	Transfusion %
Aron et al. [22]	19/19	4.2	972 mm^2^	24	74	5	89/94^a^	5
Boormans et al. [23]	23/26	7.5	6 cm^2^	18	8	8	58/81^a^	4
Dawaba et al. [24]	65/72	5.9	260 mm^2^	30	3	6	86/93^a^	1
Gonen et al. [25]	31/31	10.4	929 mm^2^	30	52	6	61/68	23
Guven et al. [26]	17/20	1.8	19 mm	28	0	0	95/95	5
Kapoor et al. [27]	31/31	9.6	DNS	30	3	10	74/84	0
Mahmud and Zaidi [28]	29/30	3.8	2.35 cm	DNS	0	0	60/100^a^	6
Nouralizadeh et al. [16]	20/24	3.1	33 mm	26	0	8	79/92^a^	5
Özden et al. [29]	51/53	9.7	654 mm^2^	30	40	6	74/87^a^	17
Rizvi et al. [30]	62/62	DNS	4.7 cm	22	DNS	0	68/95^a^	25
Salah et al. [21]	135/138	8.9	507 mm^2^	DNS	DNS	DNS	DNS/99	1
Zeren et al. [31]	55/67	7.9	283 mm^2^	30	DNS	11	DNS/87	24
